# A critical assessment framework to identify, quantify and interpret the sources of uncertainty in cost-effectiveness analyses

**DOI:** 10.1186/s12913-022-08214-9

**Published:** 2022-06-25

**Authors:** Gergő Merész, Veronika Dóczy, Áron Hölgyesi, Gergely Németh

**Affiliations:** 1 National Institute of Pharmacy and Nutrition, Szabolcs street 33, Budapest, 1135 Hungary; 2 Semmelweis University, Üllői street 26, Budapest, 1085 Hungary; 3 National Insititute of Health Insurance Management, Váci street 73/A, Budapest, 1139 Hungary

**Keywords:** Technology Assessment, Biomedical (MeSH unique ID: D013673), Decision Making, Organizational (MeSH unique ID: D003659), Evidence-Based Medicine (MeSH unique ID: D019317), Uncertainty (MeSH unique ID: D035501), Prostatic Neoplasms, Castration-Resistant (MeSH unique ID: D064129)

## Abstract

**Background:**

Using a standardized approach to describe the sources of uncertainty in cost-effectiveness analyses might bring added value to the local critical assessment procedure of reimbursement submissions in Hungary. The aim of this research is to present a procedural framework to identify, quantify and interpret sources of uncertainty, using the reimbursement dossier of darolutamide as an illustrative example.

**Methods:**

In the procedural framework designed for the critical assessment of cost-effectiveness analyses, the quantifiability of an identified source of uncertainty is assessed through the input parameters of the originally submitted model, which is followed by the interpretation of its impact on estimates of costs and outcomes compared to the base case cost-effectiveness conclusion.

**Results:**

Based on our experiences with the recent reimbursement dossier of darolutamide, the significant and quantifiable sources of uncertainty were the time horizon of the economic analysis; the restriction of the efficacy analysis population; long-term relative effectiveness of darolutamide; price discount on subsequent therapies. We identified resource use patterns for comparator and subsequent therapies as a quantifiable, yet non-significant source of uncertainty. The EQ-5D value set used to estimate utility values was identified as a non-quantifiable and potentially not significant source of uncertainty.

**Conclusions:**

The procedural framework, demonstrated with an example, was sufficiently flexible and coherent to document and structure the sources of uncertainty in cost-effectiveness analyses. The full-scale use of this framework is desirable during the decision-making process for reimbursement in Hungary. The further formalization of identifying sources of uncertainty is a possible subject of methodological development.

## Background

The critical assessment of reimbursement dossiers has been a core component of the local health technology assessment (HTA) procedure in Hungary since its institutionalisation. In order to assess the clinical and economic aspects of a pharmaceutical product, market authorisation holders are required to submit a reimbursement dossier. In addition to administrative information, the dossier includes a summary of the available clinical data, a cost-effectiveness analysis of the technology and a budget impact analysis. An economic model must also be submitted, if it was used to conduct the cost-effectiveness analysis. The legal requirement to submit the analyses is set out by a ministerial decree [1]. The local recommendations for conducting health economic assessments are set out in a methodological guidance [2]. The market authorisation holder prepares the analyses alongside the recommendations of the guidance; the local HTA body is expected to validate the submitted economic analyses to the same recommendations through the critical assessment procedure.

The critical assessment procedure, although conducted heuristically, concludes on identifying the sources of uncertainty that might alter the overall conclusion on relative effectiveness and cost-effectiveness. An ISPOR-SMDM Modeling Good Research Practices report [3] defines uncertainty as either stochastic uncertainty, parameter uncertainty, heterogeneity or structural uncertainty. The concept of stochastic uncertainty relates to the random variability in events, whereas parameter uncertainty raises from the estimation of the parameter and can be described through measures of dispersion. In contrast, heterogeneity is explained by certain characteristics of the patient population and can be depicted through subgroup analyses. The impact of parameter uncertainty on cost-effectiveness estimates can be captured via deterministic and probabilistic sensitivity analyses. A rather extensive, yet sophisticated approach to the assessment of structural uncertainty is applying calibration methods to assess the relation of input parameters to model outputs. The local guidelines for health economic assessments strongly recommends the preparation of subgroup analyses, deterministic and probabilistic sensitivity analyses as well as reporting the structural assumptions [2] to describe the respective type of uncertainty.

Several, widely used checklists (for example, CHEERS [4] and CASP [5]) are available for providing a conclusion on the quality of economic analyses, as well as to explore the structural assumptions in the economic analysis. These checklists may not always fit the needs of local HTA bodies and payers, as they do not attempt to conclude on the quantifiability or the significance of an identified methodological issue, but optimised for providing a detailed scientific conclusion on the quality of a cost-effectiveness analysis. Moreover, the structure and detail of clinical- and economic assessments in the submitted reimbursement dossiers can differ from scientific reporting standards. An adapted methodological checklist for economic assessments is available particularly for local use in Hungary [6]. Although the original aim of developing the checklist was to describe the methodological issues of economic analyses, the negative answers for each question can be used to broadly identify sources of uncertainty. In conclusion, the checklist helps to get a qualitative picture on uncertainty, but does not provide an insight into the comparable impact of each contributing source to the overall uncertainty.

Therefore, a more practical, flexible and scalable framework is needed to complement the tools of the local HTA body in formulating the conclusion on the submitted economic assessments. The comprehensive and consistent analysis of uncertainties can have a potentially high added value both for the payer and the policy maker, as this can reflect the risks that need to be managed during the reimbursement procedure. Market authorisation holders may make assumptions for their economic assessment or pick input values for model parameters that support the cost-effectiveness of a new treatment at the expense of introducing additional uncertainty. These assumptions and input values rarely make an analysis irrelevant or unrealistic, but their appropriateness depends heavily on the context and the availability of evidence. In theory, sensitivity analyses carried out by the author of the economic assessment should be sufficient to describe uncertainty. In practice, the detail and interpretation of these sensitivity analyses can be heterogeneous: if the choice of input variables and their respective input values is selective in order to support cost-effectiveness arguments, the validity of such sensitivity analyses may be restricted as well. This could be offset by using relevant, well-reasoned, but more conservative assumptions and input values for assessing uncertainty. Therefore, sensitivity analyses carried out by the HTA body on submitted economic analyses should be extended to identify, quantify and interpret sources of uncertainty to better frame the decision problem itself and potentially improve the timeliness of a decision. Eventually, appropriately described sources of uncertainty can also help in allocating the right amount of capacity to analysing them throughout decision-making.

The steps of identifying the sources of uncertainty might be difficult, but a methodological guidance of EUnetHTA [7] on the critical assessment of economic evaluations provides an appropriate basis for finding the potential sources. The framework described below is based on this guidance, and elaborates on the local checklist published earlier by distinguished authors [6].

The aim of the current research is to describe the framework for identifying, quantifying and interpreting the sources of uncertainty taking the submitted reimbursement dossier of darolutamide as an example, a second-generation androgen receptor antagonist combined with androgen-deprivation therapy for the treatment of patients suffering from nonmetastatic, castration-resistant prostate cancer.

## Methods

**Fig. 1:**
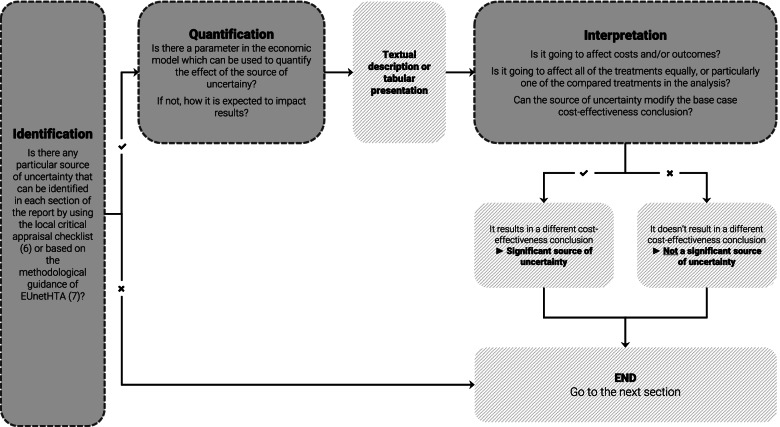
The graphical illustration of the procedural framework

The procedure set out by the framework relies on identifying, quantifying and interpreting the sources of uncertainty, and preceded by the assessment of model face validity. Therefore, the economic model and the presented base case is already deemed as suitable for supporting the economic evaluation of the technology. The procedure is illustrated in Fig. 1.

The development of the framework was initiated by reviewing the local checklist and methodological guidance mentioned above; this was followed by several internal discussions and pilots within the Department for Health Technology Assessment at the National Institute of Pharmacy and Nutrition, as well as the presentation of case studies. Feedback on the pilot application of the framework was collected via personal discussions with the relevant colleagues of the National Institute of Health Insurance Fund Management.

Within the current framework, the potential sources of uncertainty are examined according to the structure (i.e. sections on economic domains) of the critical assessment report, these are: type of economic analysis; evaluation of the economic model and its core assumptions, evaluation of economic model inputs (particularly transition probabilities, health utilities, resource use and unit costs), results and the sensitivity analyses. The tools for identifying the sources of uncertainty are the aforementioned EUnetHTA guidance [7] and the checklist developed by Inotai et al. [6]: the sections of the guidance and the topics of checklist broadly correspond to the structure of the critical assessment report structure. Although to some extent, identification also relies on expertise accumulated via the Department’s internal knowledge repository. There might be multiple sources of uncertainty identified for each section, as well as none.

After identifying the potential sources of uncertainty, the analysis of quantifiability follows. An identified source of uncertainty is considered quantifiable, if there is a relevant (and editable) parameter to depict its impact in the submitted health economic model. For each source of uncertainty, the alternative input value for the relevant model parameter is chosen based on its availability (i.e. the alternative value had already been included in the model by the applicant, but was not part of the submitted base case). It is also evaluated if all possible or relevant scenarios are considered for the particular parameter. If a more reasonable input value is identified during critical appraisal, the analyst may include it instead of the ones already available in the model. If there is no appropriate parameter in the model to quantify the impact of a source of uncertainty, and it is not feasible to implement one, it is not possible to quantify the impact of the given uncertainty. In such cases, it can be considered whether the analyst has access to information that can be used to quantify the impact of the given uncertainty.

The interpretation of all identified (both quantifiable and not quantifiable) sources of uncertainty should cover whether each of these affect.

1. Costs and/or outcomes;
2. All of the treatments equally, or particularly one of the compared treatments in the analysis.

As a final step of interpretation, the significance of the impact on cost-effectiveness results is determined. In case of a quantifiable source of uncertainty, the impact is deemed ‘significant’ if the scenario analysis with the alternative set of inputs results in the change of the conclusion on cost-effectiveness, compared to the base case. If the scenario analysis with the alternative set of inputs yields the same conclusion on cost effectiveness as in the base case, the impact of the given source of uncertainty is deemed ‘not significant’. If the source of limitation cannot be quantified within the economic model, the exact significance cannot be determined neither, so the expectations of the analyst (‘probably significant’ / ‘probably not significant’) carrying out the critical assessment can be presented as an indicative impact when interpreting the results.

The textual and tabular description of the analysis, similarly to the current research, should be presented, namely, the sources of uncertainty, their quantification and interpretation. (Table 1).

**Table 1:** Summary of the identified sources of uncertainty in the economic analysis

	Quantifiable?	Scenario available in the model?	Incremental costs (HUF)^a^	Incremental effectiveness (QALY)^a^	ICER (HUF/QALY)^a^	Impact on cost-effectiveness conclusion^b^
Base case (darolutamide is cost-effective to ADT)	-	-	ref	ref	ref	-
Time horizon of the analysis (BC: time horizon ins 27 years; ScA: time horizon is 10 years) [Type of economic analysis]	Yes	Yes	-2%	-32%	45%	Significant
Restriction of the efficacy analysis population to mITT (BC: censor patients who develop metastasis before starting treatment; ScA: patients who develop metastasis before starting treatment count as events) [Evaluation of the economic model—transition probabilities]	Yes	Yes	13%	8%	4%	Significant
Long-term effectiveness of darolutamide on overall survival (BC: assume benefit in mortality over the entire analysis time horizon; ScA: do not assume benefit in mortality after 10 years) [Evaluation of the economic model—transition probabilities]	Yes	Yes	< 1%	-5%	6%	Significant
Resource use patterns (comparator and subsequent therapies) (BC: assume equal distribution of degarelix, goserelin, leuprorelin, triptorelin and buserelin as part of ADT; ScA: differentiate the distribution of compunds used as part of ADT: higher share for degarelix, goserelin and leuprorelin, lower for triptorelin and buserelin) [Evaluation of the economic model – cost inputs]	Yes	Yes	< 1%	-	< 1%	Not significant
Price discount on subsequent treatments (BC: use the public list prices of abiraterone-acetate, enzalutamide, degarelix; ScA: assume 30% discount on abiraterone-acetate, enzalutamide, degarelix list prices) [Evaluation of the economic model – cost inputs]	Yes	No	7%	-	7%	Significant
EQ-5D value set used to estimate utilities (BC: use the UK value set when estimating utilities; ScA: use the Hungarian value set for estimating utilities) [Evaluation of the economic model – utility inputs]	No	No	-	?↕	?↕	Not quantifiable

For non-quantifiable sources of uncertainty, „?↕” marks the uncertain direction of impact. ﻿For each source of uncertainty, parentheses hold the parameter input for base case (BC) and the scenario analysis (ScA), respectively; brackets hold the name of the relevant section of the assessment report

^a^The quantifiable or expected impact on base case results of each source of uncertainty

^b^If considering the impact of the source of uncertainty changes the base case cost-effectiveness conclusion, it is deemed significant

## Results

Each of the identified sources of uncertainty are described below, containing a brief presentation of the submitted base case for each identified source, the alternative input value for the quantification of the impact, and finally, the interpretation of the results.

Table 1. provides a summary of the results. For context, the submitted base case cost-effectiveness results indicated darolutamide generating an incremental health gain of 1.31 QALYs alongside incremental costs over ADT. However, the calculated ICER is marginally below to the local cost-effectiveness threshold. For incremental costs, effectiveness and cost-effectiveness ratio, cell percentages show the deviation from the base case when the alternative input was applied in the economic model. For non-quantifiable parameters, the expected direction of deviation is included in the cell.

### Time horizon of the analysis

According to the local guideline, the time horizon of cost-effectiveness analyses should be lifelong; the base case value in the current analysis was 27 years, with the average age at baseline being 73.6 years in the ARAMIS trial [8]. However, as the life expectancy in the local male population is 10.10 years [9], the critical assessment concluded that a shorter time horizon would be reasonable for the analysis. The alternative input of 10 years was selected for the model parameter to quantify the potential impact of this source of uncertainty, affecting the costs and outcomes of both treatments compared in the analysis. This yielded a different conclusion on cost-effectiveness than in the base case therefore it’s impact was considered as significant.

### Restriction of the efficacy analysis population to mITT

In the base case of the cost-effectiveness analysis, efficacy inputs were estimated based on a modified intention-to-treat (mITT) sample of study subjects [10]. That is, patients who developed metastasis at baseline, after randomization, but before receiving the first dose of treatment were censored. To quantify the impact of this source of uncertainty, the already built-in model settings were adjusted to use the estimates on the intention-to-treat population. Using the alternative input implied the change in estimates of costs and outcomes for both treatments. This also resulted in a different conclusion on cost-effectiveness than in the base case so it’s impact was interpreted as significant.

### Long-term effectiveness of darolutamide on overall survival

Although the latest results on overall survival included in the economic model from the ARAMIS trial showed a statistically significant benefit for darolutamide (hazard ratio for death, 0.69; 95% CI, 0.53 to 0.88; *p* = 0.003), the data is still immature to draw final conclusions on the benefit in mortality risk [10]. In order to quantify the impact of this uncertainty on cost-effectiveness estimates, an already built-in model parameter was used to assume the same mortality risk for all treatment arms in the model after 10 years as an alternative input, instead of assuming an effect during the entire time horizon. Quantifying the source of uncertainty affected the effectiveness estimates for darolutamide; as the conclusion on cost-effectiveness also changed with the ICER exceeding the threshold, the impact of considering the uncertainty can be also interpreted as significant.

### Resource use patterns (comparator and subsequent therapies)

The comparator for darolutamide + ADT in the cost-effectiveness analysis was ADT alone; however, the distribution of compounds included in ADT was based on undisclosed expert opinion. By base case, an equal distribution (20% for each) of compounds was applied; this was changed to reflect a higher share for degarelix, goserelin and leuprorelin (30% for each) and a lower share for triptorelin and buserelin (5–5%). Applying the alternative input for the already available model parameter affected the cost estimates for both treatments compared in the analysis, yet the cost-effectiveness conclusion remained the same, and so its impact can be interpreted as not significant.

### Price discount on subsequent treatments

The targeted database review during the critical assessment procedure identified several risk-sharing agreements [11] and successful procurement notices [12, 13] that may impact the net prices of compounds used throughout the treatment sequence. As the impact of these agreements on prices were originally not explored in the cost-effectiveness analysis, as an alternative input for quantification, 30% price reduction was assumed for the list prices of enzalutamide, abirateron and degarelix. Adding the parameter for price discount was a minor modification to the model. The cost estimates changed for both treatments, and the resulting ICERs also yielded a change compared to the base case cost-effectiveness conclusion, so the impact of this source of uncertainty was determined significant.

### EQ-5D value set used to estimate utilities

The utility values applied in the economic model were derived based on the EQ-5D data collected in the ARAMIS clinical trial (and from some secondary sources), and UK tariffs were used to estimate the weights. However, a local tariff set, suitable to provide more relevant estimates of utility had already been published when the economic evaluation was carried out [14]. This source of uncertainty is impossible to quantify without accessing the patient-level data from the clinical trial, yet it could alter the effectiveness estimates for both treatments. Nevertheless, without the ability to quantify its impact or describe its direction, the effect of the uncertainty was described as „not quantifiable”.

## Discussion

In order to aid decision-making and ensure the relevance of their reports, national HTA bodies use different approaches in their processes to evaluate the sources of uncertainty. The procedure presented hereby is similar to the one applied by the National Institute for Health and Care Excellence. An Evidence Review Group (ERG) is commissioned to carry out complimentary economic analyses (or even to set up a de novo cost-effectiveness assessment) to identify the potential sources of uncertainty; some author suggest that the analyses of the ERG are highly influential on the outcome of the decision-making process [15]. Applying the exact same procedure would be quite challenging due to resource constraints, as the local HTA body with a headcount of 8﻿ medical and 8 economic assessors in Hungary has 50 days to carry out the critical assessment, whereas the number of pharmaceutical submissions per annum exceeded 130 in the last three calendar years.

The strength of the current research is that it provides a detailed description of the framework to identify, quantify and interpret sources of uncertainty in economic analyses which is highly relevant in the local decision-making process. While building on past research on a methodological checklist and the experience gained in international cooperation, the presented framework operationalises the critical assessment of economic evaluations as well as contributes to the standardisation of local critical assessment reports.

Good practices on approaching uncertainty in economic assessments have been distilled in recent years. The report of Fenwick and colleagues [16] demonstrates value of information (VoI) analysis assessing the extent to which the information generated through research on a particular parameter would improve the expected payoffs associated with a decision by reducing the uncertainty surrounding it. Nevertheless, the concept of VoI focusses on interpreting parameter uncertainty, whereas the framework presented hereby enables the explicit evaluation structural uncertainty, prompting potential synergy for the complex evaluation of quantifiable and significant sources of uncertainty.

However, this framework has a number of limitations. First, we only discussed one economic analysis of a pharmaceutical product as a test case in this paper, which can potentially limit the generalisability of our findings. Nevertheless, more experience is expected with its full implementation in the local assessment procedure. The current research does not provide a trivial methodology to further operationalise the identification of sources of uncertainty, nor to assess the identified, yet non-quantifiable sources of uncertainty. Although we did not manage to provide an approach to fully capture these aspects of evaluating uncertainty, these both would be highly desirable in order to ensure that the critical assessment of rather heterogeneous economic analyses are consistently presented in a structured fashion. The price of the technology is for particular interest when ensuring the cost-effectiveness or purchasing, but the current analysis does not entirely cover this source of uncertainty, as this is usually managed through confidential price negotiations between the payer and the market authorisation holder, which take place after closing the assessment. A new, alternative cost-effectiveness estimate considering the mutually exclusive sources of uncertainty is frequently provided as an outcome of the critical assessment process: however, we did not discuss this as part of the current research. The interpretation of significance indirectly relies on the base case value itself: as seen in the current example, if the base case ICER is close to the cost-effectiveness threshold, a small change in its absolute values may yield a high number of sources with a significant impact on the cost-effectiveness conclusion. The impact of this limitation can be mitigated: given that an economic assessment is considered credible, a base case ICER well below the willingness-to-pay threshold yields a lower risk for arriving at a false cost-effectiveness conclusion, even if a remarkable source of uncertainty is identified. The full presentation of the base case cost-effectiveness results for darolutamide would have been advantageous, but due to local confidentiality rules, the incremental costs of the intervention had to be omitted, as this can be informative of the offered list price before the formal decision on reimbursement. Finally, the current research does not cover the sources of uncertainty that might have a significant impact on the cost-effectiveness of a technology (such as unit costs of health services), but for which the uncertainty is difficult to quantify due to the local health financing environment.

## Conclusions

In this research, we have demonstrated the feasibility of the framework for identifying, quantifying and interpreting the sources of uncertainty through the critical assessment of an economic evaluation. In our view, this framework is a methodological development, which complements the already existing tools for critically assessing the submitted reimbursement dossiers. Therefore, the framework is suitable to describe and assess the sources of uncertainty in economic analyses submitted for reimbursement purposes in Hungary. The application of the framework in the daily routine of the reimbursement procedure may increase the efficiency of decision-making. A potential improvement of the framework is to further formalise the identification of sources of uncertainty; increasing the transparency of documents used during the critical assessment process in general is also desirable to facilitate external quality control.

Given that a similar description operationalising and standardising the critical assessment procedure is not available now, we firmly believe that our work contributes to taking the critical assessment process in Hungary to the next level.

## Data Availability

All data generated or analysed during this study are included in this published article.

## Abbreviations

ADT: Androgen Deprivation Therapy
CHEERS: Consolidated Health Economic Evaluation Reporting Standards
CASP: Critical Appraisal Skills Programme
EUnetHTA: European Network for Health Technology Assessment
ERG: Evidence Review Group
HTA: Health Technology Assessment
ICER: Incremental Cost-Effectiveness Ratio
mITT: Modified Intention-to-Treat
QALY: Quality-Adjusted Life Year﻿s
VoI: Value of Information Analysis
